# Modulation by Cocaine of Dopamine Receptors through miRNA-133b in Zebrafish Embryos

**DOI:** 10.1371/journal.pone.0052701

**Published:** 2012-12-21

**Authors:** Katherine Barreto-Valer, Roger López-Bellido, Fátima Macho Sánchez-Simón, Raquel E. Rodríguez

**Affiliations:** Department of Biochemistry and Molecular Biology, Institute of Neuroscience of Castilla y León, University of Salamanca, Salamanca, Spain; Florida State University, United States of America

## Abstract

The use of cocaine during pregnancy can affect the mother and indirectly might alter the development of the embryo/foetus. Accordingly, in the present work our aim was to study *in vivo* (in zebrafish embryos) the effects of cocaine on the expression of dopamine receptors and on miR-133b. These embryos were exposed to cocaine hydrochloride (HCl) at 5 hours post-fertilization (hpf) and were then collected at 8, 16, 24, 48 and 72 hpf to study the expression of dopamine receptors, *drd1, drd2a, drd2b* and *drd3*, by quantitative real time PCR (qPCR) and in situ hybridization (ISH, only at 24 hpf). Our results indicate that cocaine alters the expression of the genes studied, depending on the stage of the developing embryo and the type of dopamine receptor. We found that cocaine reduced the expression of miR-133b at 24 and 48 hpf in the central nervous system (CNS) and at the periphery by qPCR and also that the spatial distribution of miR-133b was mainly seen in somites, a finding that suggests the involvement of miR-133b in the development of the skeletal muscle. In contrast, at the level of the CNS miR-133b had a weak and moderate expression at 24 and 48 hpf. We also analysed the interaction of miR-133b with the Pitx3 and Pitx3 target genes *drd2a* and *drd2b,* tyrosine hydroxylase (*th*) and dopamine transporter (*dat*) by microinjection of the Pitx3-3'UTR sequence. Microinjection of Pitx3-3'UTR affected the expression of *pitx3*, *drd2a, drd2b, th* and *dat*. In conclusion, in the present work we describe a possible mechanism to account for cocaine activity by controlling miR-133b transcription in zebrafish. Via miR-133b cocaine would modulate the expression of *pitx3* and subsequently of dopamine receptors, *dat* and *th*. These results indicate that miRNAs can play an important role during embryogenesis and in drug addiction.

## Introduction

The use of cocaine during pregnancy can affect embryonic or foetal development. Owing to its small size, the cocaine molecule can easily cross the placental barrier and enter the foetal blood system [1], producing a high risk of abortion, stillbirth, premature delivery, placental abruption, babies with low birth weight, as well as abnormalities in the liver, lungs, genitals and heart system [2], [3], [4], and in the nervous system [5], [6], [7], [8], [9], including changes in the dopaminergic signalling mechanism in the foetal brain [10], [11], [12], [13]. Moreover, several studies in humans and in animal models have confirmed that prenatal exposure to cocaine results in permanent changes at the behavioural, cellular and molecular levels [14], [15], [16].

Animal models using mammals (rat, mouse, rabbits and chimpanzees) have been developed to elucidate the mechanism by which cocaine induces addiction [10], [11], [12], [13], but at present no clear pathways have been proposed to explain cocaine addiction and hence no effective treatment against this adverse effect is available. In recent years, the use of zebrafish has grown considerably as a powerful animal model for studying molecular dopamine receptors [17], [18], [19], and actions of cocaine at the molecular and behavioural levels [20], [21] owing to their similarity to mammals [22]. Zebrafish also have the advantage that during their development they develop rapidly, are transparent, permeable to drugs and can be manipulated easily using genetic and molecular approaches [23], [24]. Eight dopamine receptors in the zebrafish have been cloned: Drd1, which shares 71% amino acid identity to humans [17]; Drd2a, Drd2b, Drd2l, Drd3, Drd4a, Drd4b and Drd4rs, which show a homology of between 56 and 67% as compared to the human sequence [18], [19]. Additionally, zebrafish express Pitx3, which is a transcription factor that positively regulates the synthesis of tyrosine hydrolase (TH), the vesicular monoamine transporter, the dopamine transporter (DAT), and the Drd2 receptor [25].

Another important point about the effects of cocaine during early development is that there are few studies available (either in mammals or in zebrafish). Zebrafish-cocaine studies have shown that exposure to cocaine at concentrations comparable to those experienced by human embryos (0.3 µM) can acutely alter the expression of circadian genes (melatonin signalling, growth and neurotransmission), while repeated exposure to cocaine can alter neuronal development [20]. It has also been described that zebrafish larvae are sensitive to cocaine. Thus, while low doses (0.2, 0.6 and 1.9 µM) do not produce changes in locomotor activity, high doses (5.6, 16.7 and 50 µM) reduce locomotor responses in a similar way as described for mammals [26]. Behavioural studies in adult zebrafish have also shown that zebrafish develops cocaine-induced place preference [27] and that cocaine withdrawal can produce an anxiety-like state [21].

Recently, many authors have reported that small non-coding RNAs known as microRNAs (miRNAs or miRs) bind to complementary sites in the 3'UTR region of mRNA and can therefore disrupt the expression and degrade and/or inhibit the translation of mRNA to amino acids [28], [29], [30]. In mammals, Pitx3 transcription is regulated by miRNA 133b (miR-133b), which inhibits the differentiation of midbrain dopaminergic neurons from embryonic stem cells [31]. Emerging evidence has revealed that miRNAs are also present in the CNS [32], [33], where cocaine administration alters the expression of many miRNAs (let-7d, miR-1, miR-124, miR-181a, miR-29b, miR-31, miR-382 and miR-212) in brain regions related to cocaine addiction (nucleus accumbens, ventral tegmental area, prefrontal cortex and dorsal striatum) [34], [35], [36]. Cocaine-induced synaptic plasticity in dopamine neurons might play a role in the development of drug addiction (by altering the fine tuning of dopaminergic cell firing) [37], [38], [39], [40], and since miRNAs are involved in synaptic plasticity [36], [41], [42] the study of miRNAs in the field of addiction could answer the question of how cocaine produces addiction. Recently, it has been reported that overexpression of miR-212 protects against the development of cocaine addiction [35], pointing to a crucial role of miRNAs in the addiction process.

Taking all the above into consideration, it is evident that the zebrafish is a good model for the study of cocaine during embryonic developmental process. Accordingly, we surmised that exposure to cocaine during the early stages of embryonic development in zebrafish could induce changes in the expression of genes related to the addiction process such as dopamine receptors, DAT and TH. Since these genes are targets of the transcription factor Pitx3, and this latter is also target of miR-133b, it is likely that cocaine via miR-133b modulates the expression of dopamine receptors, *dat* and *th*.

In this investigation we report that embryos exposed to cocaine in the blastula stage (5 hpf) of zebrafish embryos show alterations in the expression of the dopamine receptors (*drd1, drd2a, drd2b* and *drd3*) and *pitx3*; these changes caused by cocaine would be via miR-133b. Such changes produced by cocaine resemble the actions of cocaine in mammalian models, showing that the zebrafish is a suitable model for the study of gene expression caused by cocaine, since the molecular and phylogenetic analysis of dopamine receptors are very similar in comparison to its mammalian counterparts. Moreover, the action of cocaine on miR-133b, related to the development of the dopaminergic system, indicates that this miRNA could play an important role in drug addiction. Thus, as a model to study the actions of cocaine during development zebrafish can provide essential insight into of the addiction process and in the future permit us to find an effective treatment against cocaine addiction.

## Results

### Doses-effects of Cocaine

In order to determine the optimal dose that produces differences in the changes of gene expression we exposed doses of 0.5, 1.5 and 3 µM cocaine HCl at 5 hpf of development of the zebrafish embryos. Gene expression was analysed at 24 hpf. 1.5 µM cocaine HCl induced substantial changes in gene expression of most genes studied (miR-133b, *pitx3, dat, th, drd2a* and *drd2b*) in comparison to the control group. The higher concentrations did not show additional effects respect 1.5 µM cocaine HCl (Figure S1). Therefore, in the present study we employed the dose of 1.5 µM cocaine HCl.

### Amount of Cocaine Entering Zebrafish Embryos

Cocaine was detected in each zebrafish embryo exposed to 1.5 µM cocaine-HCl from 5 to 24 hpf and from 5 to 48 hpf. At 24 and 48 hpf, the embryos were dechorionated with the aim of determining the amount of cocaine that had passed through the chorion to the embryos. By HPLC-MS analysis, we determined that almost 0.175±0.0472 nm of cocaine had passed to each zebrafish embryo exposed to 1.5 µM cocaine-HCl from 5 to 24 hpf and from 5 to 48 hpf, which represents approximately 12% of the initial concentration of the 1.5 µM cocaine used.

### Phylogenetic Analysis of Zebrafish Dopamine Receptors

**Figure 1:**
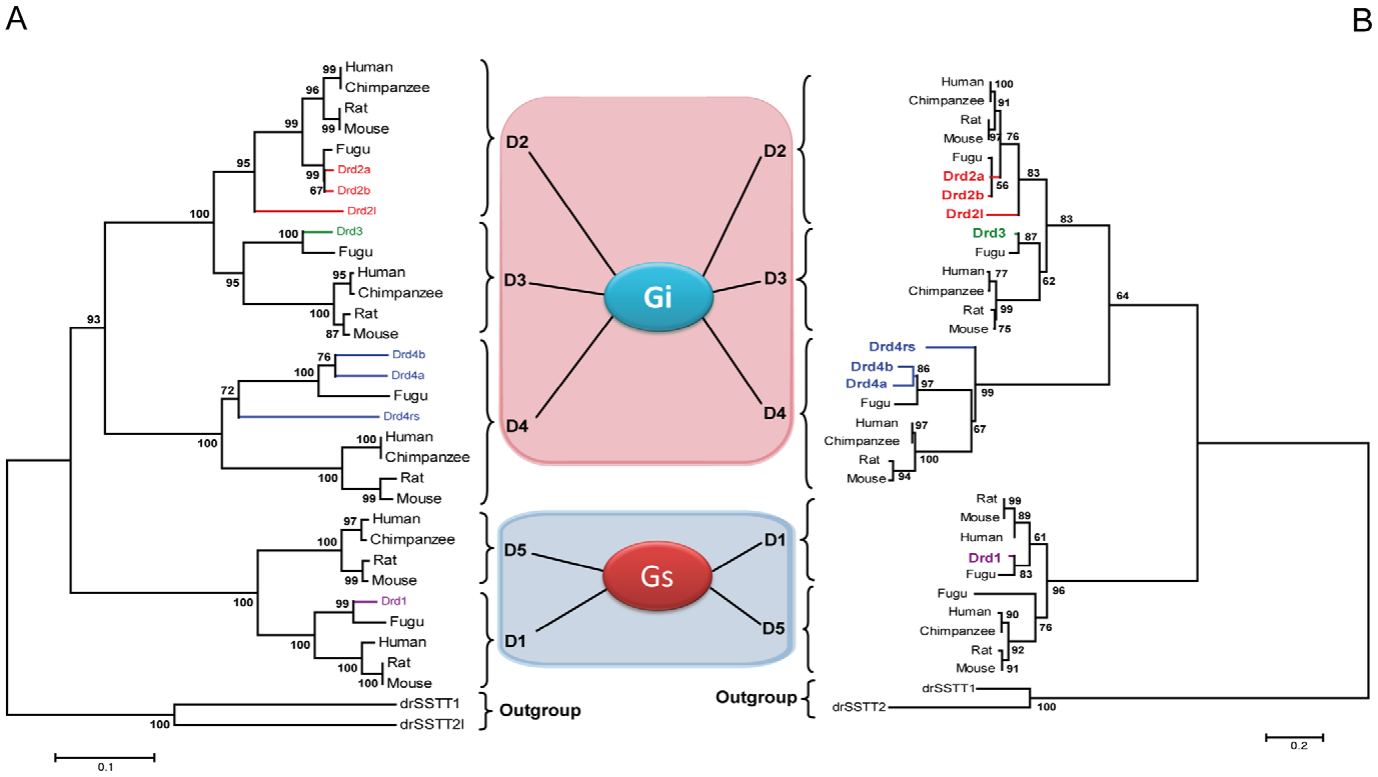
Phylogenetic analyses of vertebrate dopamine receptors in several species. The tree was routed with somatostatin receptors (drSSTT1: XP_696666.1; drdSTT2: XP_689033.4). A) The evolutionary history was inferred using the Neighbour-Joining method (NJ) [43]. The optimal tree with the sum of branch length = 2.67314359 is shown. The percentage of replicate trees in which the associated taxa clustered together in the bootstrap test (1000 replicates) is shown next to the branches as percentage values. The tree is drawn to scale, with branch lengths in the same units as those of the evolutionary distances used to infer the phylogenetic tree. Evolutionary distances were computed using the p-distance method. B) The evolutionary history was inferred using the Maximum Likelihood (ML) method [44], which shows the most probable reconstruction and representative phylogeny of the groups. The percentage of replicate trees in which the associated taxa clustered together in the bootstrap test (1000 replicates) is shown next to the branches as percentage values. Gs and Gi represent the subunit of the G protein coupled that is related to dopamine receptors. Evolutionary analyses were conducted using MEGA5 [84]. DRD1: Human DRD1 (NP_000785.1), Mouse (NP_034206.1), Rat (NP_036678.1), *Danio rerio* (NP_001129448.1), Chimpanzee (XP_001149473.2), Fugu (ENSTRUG00000011080). DRD2: Human (NP_000786.1), Mouse (NP_034207.1), Rat (NP_036679.1), Chimpanzee (NP_001029100.1), Drd2a (NP_898891.1), Drd2b (NP_922918.1), Drd2l (NP_922917.1), Fugu (ENSTRUG00000014690). DRD3: Human (NP_000787.2), Mouse (NP_031903.1), Rat (NP_058836.1), Chimpanzee (NP_001029084.1), Fugu (ENSTRUG00000000584), Drd3 (NP_898890.1). DRD4: Human (NP_000788.2), Chimpanzee (XP_001149749.1), Drd4a (NP_001012634.1), Drd4b (NP_001012636.1), Drd4rs (ENSDARG00000001912), Mouse (NP_031904.1), Rat (NP_037076.1), Fugu (ENSTRUG00000015396). DRD5: Human (NP_000789.1), Rat (NP_036900.1), Mouse (NP_038531.1), Chimpanzee (XP_528711.2).

Dopamine receptors are classified in two families: the D1-like receptor family (which consists of multiple D1 and D5 receptors) (mediated by the G_s_α, subunit of G protein) and the D2-like family (D2, D3 and D4) (mediated by G_i_α). We surmised that according to their phylogenetic alignment these zebrafish dopamine receptors would indicate which type of G protein they are coupled to (G_s_α or G_i_α). Moreover, this is the first time that an analysis of phylogeny has been made taking all zebrafish dopamine receptors together and using two types of phylogenetic analyses, the Neighbour-Joining method (NJ) [43] and the Maximum Likelihood (ML) [44] methods, to confirm that the alignment of the amino acid sequences was indeed due to the sequence of the receptor species and not to the algorithms used in the methods. Thus, to examine the evolutionary relationships between different representative species -human, chimpanzee, rat, mouse, fugu and zebrafish dopamine receptors- we conducted a phylogenetic analysis. The Drd1, Drd2a, Drd2b, Drd3, Drd4a, Drd4b and Drd4rs receptors were aligned in the clusters of their respective D1–D5 dopamine receptors in mammals, with a clear demarcation between zebrafish dopamine receptors and their orthologues (Fig. 1A and 1B). These observations were strongly supported by bootstrap analysis in NJ and ML. Moreover, zebrafish dopamine receptors formed two receptor families, where each family was associated with a specific type of G protein (G_s_α or G_i_α). The phylogenetic trees were routed with somatostatin receptors from *Danio rerio* (drSSTT1 and drSSTT2l), a member of the group of GPCRs.

### Cocaine Affects the Expression of Dopamine Receptors during Zebrafish Embryonic Development

All drugs, including cocaine, that can lead to addiction increase dopamine neurotransmitter levels in the nucleus accumbens (NAc). For instance, cocaine blocks DAT [45]. This indicates that the dopaminergic system plays a central role in the addiction process. Concerning the known dopamine receptors, it has been reported that DRD1 and DRD2 are the most involved in cocaine addiction [13], [46], whereas DRD3 and DRD4 are less relevant in the addiction process [47]. Nevertheless, the three receptors, D1, D2 and D3, have been described in regions related to cocaine addiction (prefrontal cortex, NAc, ventral tegmental area, dorsal striatum) [13], [46], [48], [49], [50]. Moreover, over the past few years zebrafish dopamine receptors have been cloned, and this has shown that these receptors are distributed in the CNS. Since zebrafish exhibit a type of behaviour similar to that observed in mammals after cocaine withdrawal, this indicates that zebrafish may be a valuable model for the study of the effects of cocaine on dopamine receptors. The above observations, and the fact that cocaine is one of the most addictive drugs that alters the functionality of the dopaminergic system, led us to study only 4 receptors- *drd1, drd2a, drd2b and drd3* - in zebrafish embryos.

**Figure 2:**
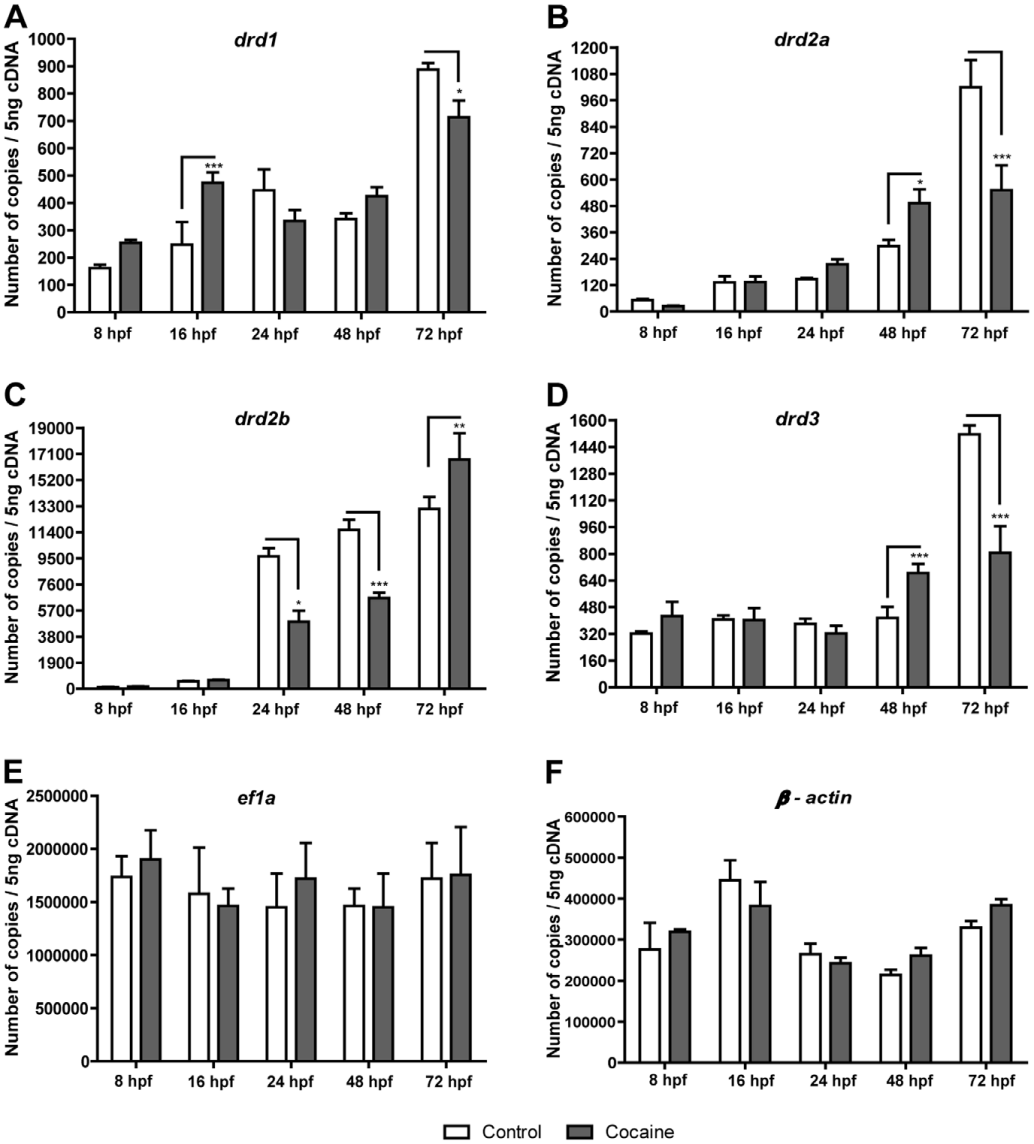
Effects of cocaine on the expression of dopamine receptors. A) *drd1*, B) *drd2a*, C) *drd2b* and D) *drd3* gene expression levels were measured at 8, 16, 24, 48 and 72 hpf using qPCR. All results were normalized to *ef1α* expression and *β-actin* (Fig. 2E and F, respectively). Two hundred and fifty embryos were used to extract RNA and synthesize cDNA. Error bars represent means of mRNA copies at each developmental stage ± SEM. Three independent experiments (each performed three times) were conducted for each stage. *P* values were calculated by two-way ANOVA, with the Bonferroni post-hoc test: *P<0.05, **P<0.01 and ***P<0.001.

**Figure 3:**
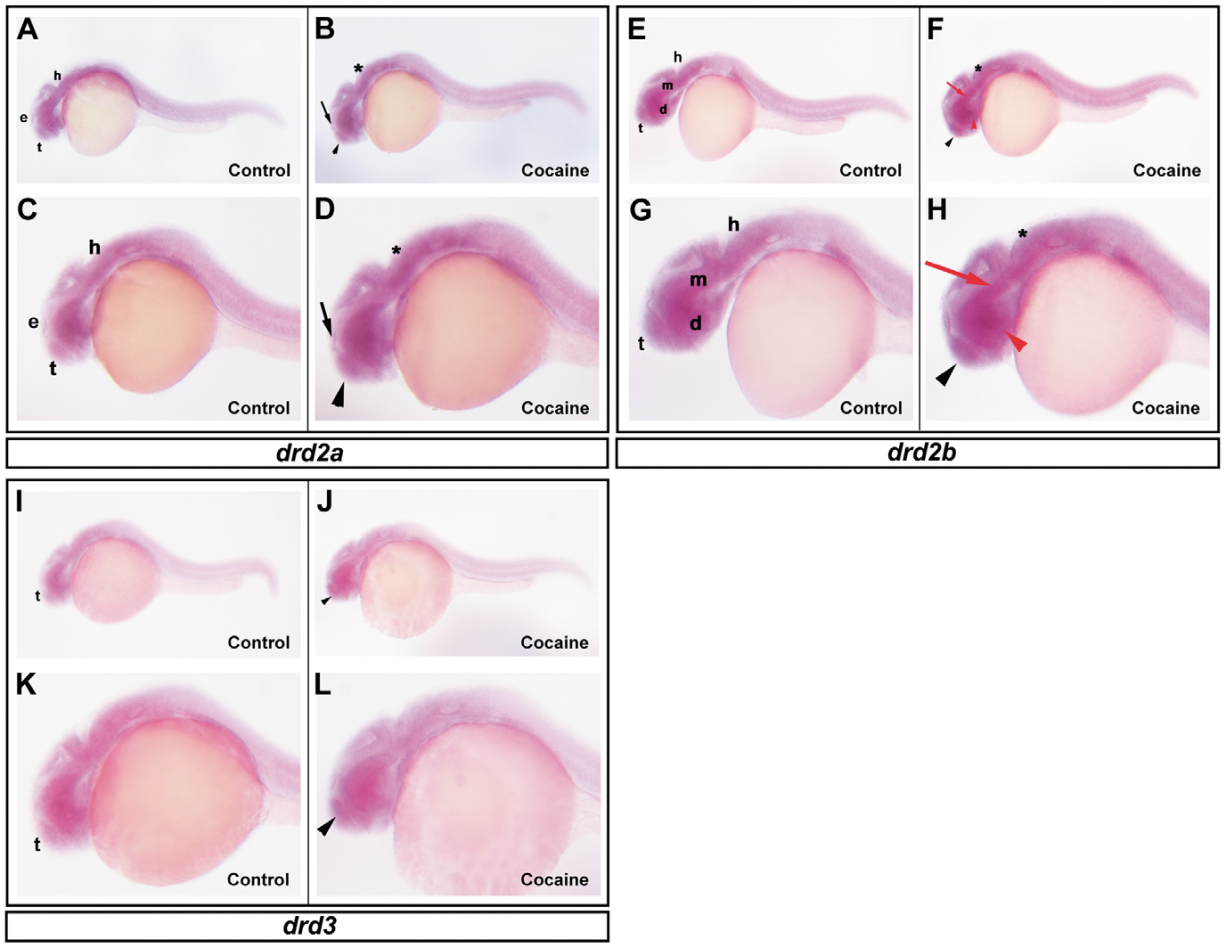
Effects of cocaine on the spatial distribution of *drd2a, drd2b* and *drd3*. Lateral view showing the expression of *drd2a* in embryos of 24 hpf. Control group (A and C) and cocaine group (B and D). *drd2a* is expressed in the epiphysis (e), tegmentum (t) and hindbrain (h) (A and C). Embryos exposed to cocaine (B and D) show an increase in the epiphysis (black arrow), tegmentum (black arrow head) and hindbrain (asterisk). Expression of *drd2b* in zebrafish embryos at 24 hpf. Control group (E and G) and cocaine group (F and H). *drd2b* is expressed in the tegmentum (t), diencephalon (d), mesencephalon (m) and in hindbrain (h) regions. Exposure to cocaine slightly increased the expression of *drd2b* in the regions mentioned (black arrow head, red arrow head, red arrow and asterisk, respectively). *drd3* expression in zebrafish embryos at 24 hpf. Control group (I and K) and cocaine group (J and L). The expression of *drd3* is seen mainly in tegmentum (t). Exposure to cocaine increased the expression of *drd3* in tegmentum (black arrow head). Scale bars = 300 µm and 6X of magnification (A, B, E, F, I and J); 250 µm and 12X magnification (C, D, G, H, K and L). d: diencephalon (red arrow head), e: epiphysis (black arrow); h: hindbrain (asterisk); m: mesencephalon (red arrow); t: tegmentun (black arrowhead).

Our results revealed that *drd1* receptor expression was increased after exposure to cocaine at 16 hpf (*P*<0.001) and decreased at 72 hpf (*P*<0.05) (Fig. 2A). Regarding *drd2a,* cocaine increased its expression at 48 hpf (*P*<0.05), while at 72 hpf the number of *drd2a* transcripts was decreased (*P*<0.001) (Fig. 2B). Conversely to *drd2a,* the *drd2b* receptor was downregulated at 24 and 48 hpf (*P*<0.05; *P*<0.001) by exposure to cocaine and was increased at 72 hpf (*P*<0.01) (Fig. 2C). Regarding the *drd3* receptor, cocaine produced an upregulation at 48 hpf, (*P*<0.001) and a downregulation at 72 hpf (*P*<0.001) (Fig. 2D). Our results thus show that cocaine alters dopaminergic receptor expression, depending on the receptor and the developmental stage. The expression of dopamine receptors was normalized by employing two different reference genes: *ef1α* and *β*-actin. According to our experiments, these genes underwent few variations in their expression at the different stages of zebrafish development (8, 16, 24, 48 and 72 hpf); none of the variations in their expression at the different stages of embryonic development were statistically significant. *ef1α* (Fig. 2E) showed a higher expression than *β-actin* (Fig. 2F) during all the development stages studied. We also studied the changes produced by cocaine in the spatial distribution of *drd2a, drd2b and drd3* at 24 hpf by ISH. The zebrafish *drd1* dopamine receptor was not taken into account since the first presence of this receptor is seen at approximately 30 hpf [17]. The expression of *drd2a, drd2b and drd3* in the CNS at 24 hpf was similar to the one described in previous study [18]. *drd2a* (Fig. 3A and C) was expressed in the epiphysis; *drd2b* (Fig. 3E and G) in the tegmentum and hindbrain, and *drd3* in the tegmentum (Fig. 3I and K). Exposure to cocaine increased the expression of *drd2a* in the epiphysis (Fig. 3B and D) and *drd2b* in the tegmentum and hindbrain (Fig. 3F and H). Additionally, the expression of *drd3* in the tegmentum was increased by exposure to cocaine (Fig. 3J and L).

### Cocaine Affects miR-133b and *pitx3* Expression

**Figure 4:**
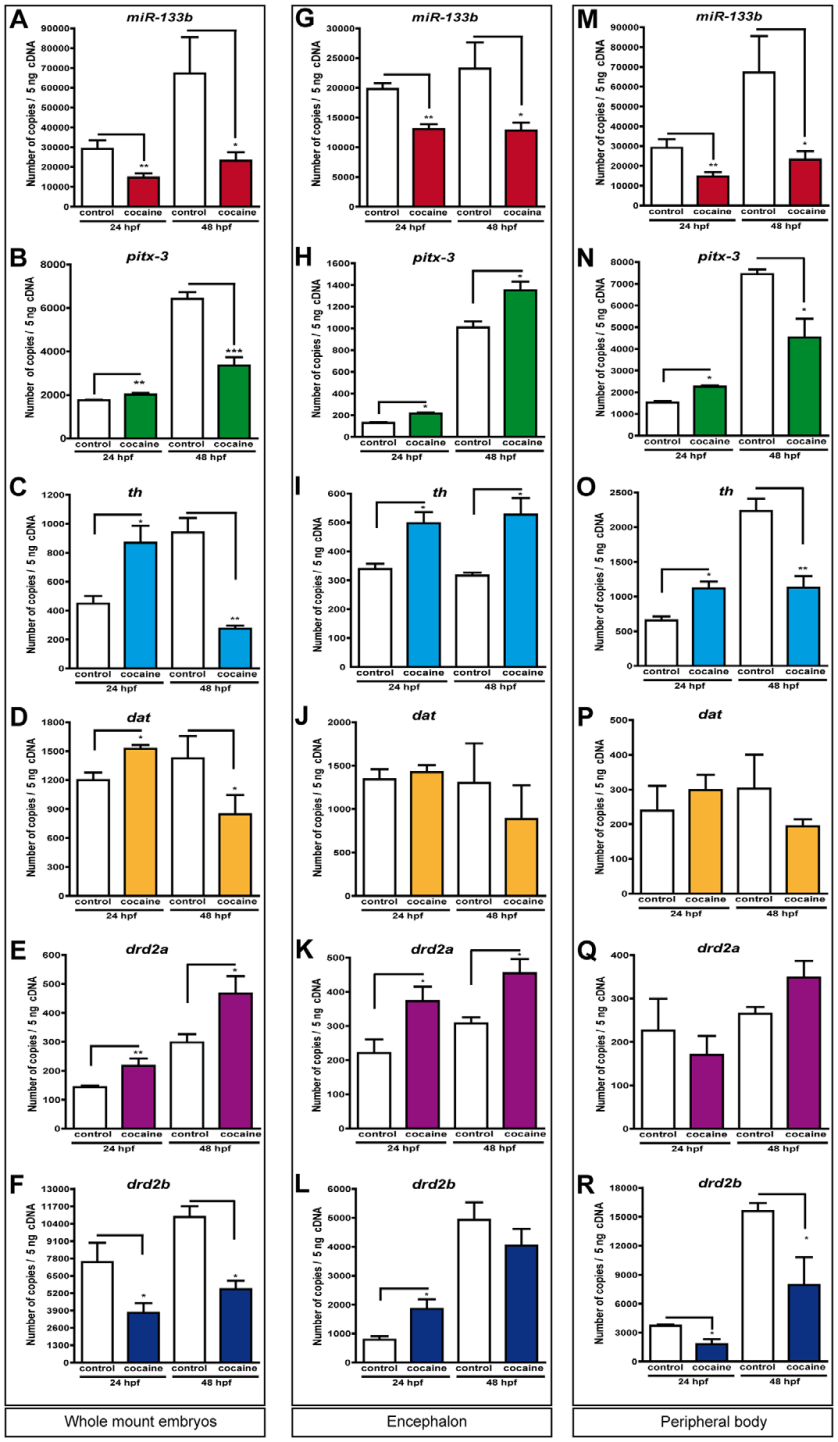
Effects of cocaine on the expression levels of miR-133b, pitx3 and its targets. Expression of miR-133b, *pitx3* and its targets genes (*th, dat, drd2a* and *drd2b*) at 24 and 48 hpf in whole-mount embryos (A–F), in the head (G–L) and at the periphery (M–R). Total RNA was isolated from two hundred and fifty embryos and used to synthesize cDNA. Gene expression levels were measured at 24 and 48 hpf by qPCR and were normalized to *ef1α* expression. Error bars represent means of mRNA copies at each developmental stage ± SEM. Data are representative of three independent experiments and each experiment was performed three times. *P* values were calculated using two-way ANOVA followed by Bonferroni's post-hoc comparisons tests: *P<0.05, **P<0.01 and ***P<0.001.

Pitx3 activates the expression of different genes such as *th, dat* and *drd2* in dopaminergic neurons [25] and it also induces miR-133b transcription by RNA polymerase II and miRNA binding to 3'UTR-Pitx3 [31], inducing mRNA degradation and blocking the translation of Pitx3. Considering the importance of miR-133b and Pitx3 in the differentiation of dopaminergic neurons, we studied the cocaine-induced changes in the expression of miR-133b and *pitx3* at 24 and 48 hpf by qPCR. Our results working with whole-mount embryos (Fig. 4A) revealed that cocaine reduced the number of miR-133b molecules at 24 and 48 hpf with respect to the control group (*P*<0.01; *P<*0.05). Moreover, cocaine produced an increase in the expression of *pitx3* at 24 hpf and, conversely, a decrease of it at 48 hpf (*P*<0.01; *P*<0.001) (Fig. 4B). The Pitx3 targets genes such as *th*, *dat* and dopamine receptor 2 were also analysed. At 24 hpf *th, dat* and *drd2a* were upregulated (Fig. 4C, D and E, respectively); at 48 hpf *th* and *dat* decreased their expression (Fig. 4C and D, respectively), whereas *drd2a* was increased (Fig. 4E). Finally, *drd2b* (Fig. 4F) exhibited different pattern of expression due to exposure to cocaine; this receptor was donwnregulated at 24 and 48 hpf.

### Cocaine Decreases miR-133b Levels in the Encephalon and at the Periphery Body

The expression of miR-133b was also analysed in the encephalon of zebrafish embryos (the embryos were decapitated, see Material and Methods) and at the periphery (the rest of the body after decapitation). The qPCR experiments showed that cocaine decreased the expression of miR-133b at 24 and 48 hpf in the head (Fig. 4G) (*P*<0.001 and *P*<0.05, respectively). When embryos were exposed to cocaine at 5 hpf and were then decapitated at 24 hpf, *pitx3* (Fig. 4H) and *th* (Fig. 4I) showed an upregulation at both 24 and 48 hpf that was statistically significant. The mRNA expression of DAT (Fig. 4J) did not show consistent changes at 24 and 48 hpf. The zebrafish dopamine receptors underwent an increase in the expression of *drd2a* in both stages (Fig. 4K) (*P*<0.05) and the expression of *drd2b* increase at 24 hpf (Fig. 4L) (*P*<0.05). At 48 hpf, no consistent changes in *drd2b* levels were observed (Fig. 4L). At the periphery, the expression of miR-133b was decreased at 24 and 48 hpf (Fig. 4M) (*P*<0.01; *P<*0.05). Cocaine produced an increase in *pitx3* (Fig. 4N) (*P*<0.05) and *th* (Fig. 4O) (*P*<0.05) levels at 24, whereas at 48 hpf it decreased the expression of *pitx3* (Fig. 4N) and *th* (Fig. 4O) (*P*<0.05; *P<*0.01). *dat* (Fig. 4P) and *drd2a* (Fig. 4Q) did not reveal consistent changes induced by cocaine at 24 or 48 hpf. Expression of the duplicated *drd2b* was downregulated at 24 and 48 hpf (in both cases *P*<0.05).

### Effects of Cocaine on the Distribution of miR-133b in Embryos at 24 and 48 hpf

**Figure 5:**
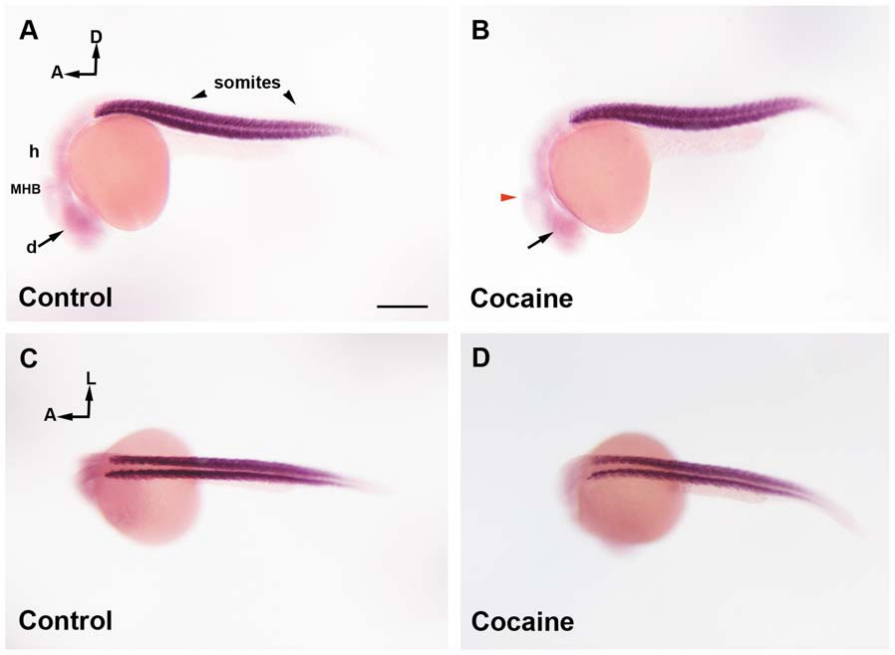
miR-133b distribution in zebrafish embryos at 24 hpf by whole-mount ISH. Control Group (A and C) and cocaine group (B and D). In a lateral view of miR-133b expression (A), the miRNA is mainly localized in somites and weakly in the brain (diencephalon, midbrain, MHB and hindbrain). Embryos exposed to cocaine (B) show a decrease in diencephalic expression (black arrow), and MHB (red arrowhead). A dorsal view of miR-133b (C) shows that this miRNA is mainly present in somites, although it is difficult to determine whether cocaine affects the expression of miR-133b in this area (D). Scale bars = 300 µm. A, B; C; D = 6X. A: anterior; D: dorsal; L: lateral; d: diencephalon; m: midbrain, h: hindbrain; MHB: Midbrain Hindbrain Boundary, sm: skeletal muscle.

**Figure 6:**
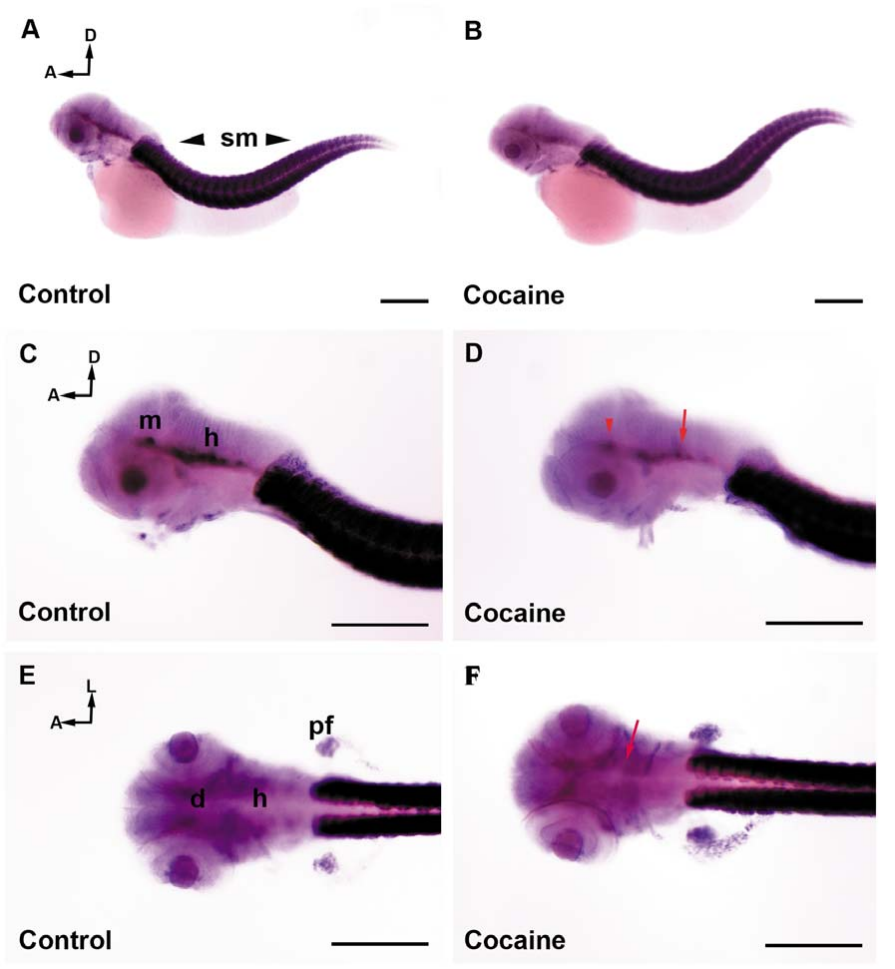
miR-133b distribution in zebrafish embryos at 48 hpf by whole-mount *in situ* hybridization (ISH). Control Group (A, C and E) and cocaine group (B, D and F). The expression of miR-133b is mostly found in the skeletal muscles (A, C and E) and to a lesser extent in the CNS (A, C and E). miR-133b is weakly expressed in the diencephalon, midbrain, and hindbrain. Embryos exposed to cocaine (B, D, and F) show a decrease mainly in the CNS (D), midbrain (red arrow head) and hindbrain (red arrow), while in the diencephalon (black arrow) the decrease less patent. The effect of cocaine on the expression of miR-133b in muscle is difficult to determine (D and F). Scale bars: 300 µm. A, B = 5X, and C, D, E, F = 10X. A: anterior; D: dorsal; L: lateral; d: diencephalon; m: midbrain; h: hindbrain; MHB: Midbrain Hindbrain Boundary; sm: skeletal muscle.

The small size of miRNA makes it difficult to detect by ISH with conventional riboprobes. Therefore, we used an LNA™ probe, which is ideal for the detection of short RNA targets. We analyzed the distribution of the miR-133b expression pattern in control and cocaine-exposed embryos at 24 (Fig. 5) and 48 hpf (Fig. 6). At 24 hpf, in control group, miR-133b was strongly detected in somites along the tail and also in the brain; mainly in the diencephalon (Fig. 5A and C). In our case, cocaine exposure at 24 hpf produced a slight decrease in the diencephalon and MHB (Fig. 5B), whereas no clear changes were observed in the somites of miR-133b (Fig. 5B and D) due to exposure to the drug. In the control embryos studied at 48 hpf, miR-133b was strongly expressed in skeletal muscle and the pectoral fins, while in the brain it was more localized in the midbrain and hindbrain, following the pathway of the median longitudinal fasciculus (Fig. 6 A, C and E). Cocaine produced a decrease in miR-133b expression in the midbrain and hindbrain (Fig. 6B, D and F). miR-133b expression in skeletal muscle seemed to be unaffected by exposure to cocaine at both 24 and 48 hpf. In order to determine the expression (qPCR) of miR-133b both in the CNS and at the periphery, we decapitated the embryos at 24 and 48 hpf with the aim of analysing the changes of miR-133b in the CNS and the periphery. We observed that miR-133b was expressed in higher amounts at the periphery (Fig. 4M) than in the CNS (4G). These results were consistent with our ISH analyses of miR-133b (Fig. 5A and 6A). Thus, the major changes observed in our qPCR experiments occurred at the periphery, although they were less intense. miR-133 also underwent changes in the CNS that were important enough to produce changes in the expression of its target gene: the transcription factor Pitx3.

The miR-133b target gene Pitx3 is expressed in the CNS and also in muscle [51], [52]. When the embryos were decapitated, the expression of *pitx3* was observed to be higher at the periphery than in the CNS, and the pattern of expression was similar to the situation observed in the whole-mount embryos. Conversely, there was only a low expression of *pitx3* in the CNS, although an increase in its expression was observed at 24 and 48 hpf. These results indicate that the changes in the expression of *pitx3* (at 48 hpf) in the CNS were masked by its expression at the periphery. In the CNS a decrease in miR-133b, induced by cocaine, at 48 hpf produced an upregulation of its target gene: the transcription factor *pitx3.* Hence, it is unclear how *pitx3* has different expression profiles in the CNS and at the periphery.

### Effects of miR-133b on the Expression of *pitx3* and its Targets

**Figure 7:**
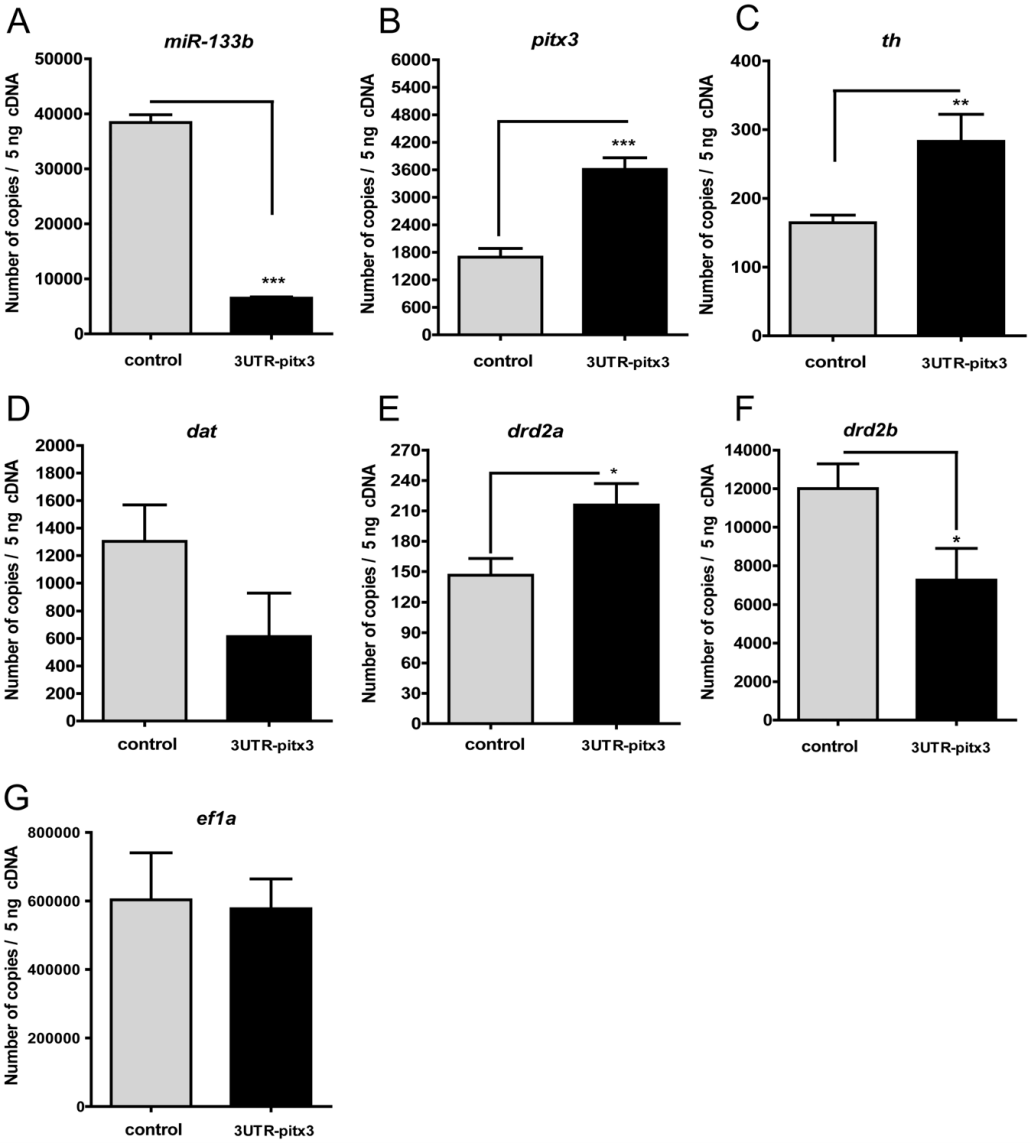
Expression of *miR-133b*, *pitx3* and its target genes (*th, dat, drd2a* and *drd2b)* in zebrafish embryos microinjected with the Pitx3-3'UTR sequence at 24 hpf. Expression of miR-133b (A), *pitx3* (B), *th* (C), *dat* (D), *drd2a* (E) and *drd2b* (F) in zebrafish embryos microinjected with the Pitx3-3'UTR. Two hundred and fifty embryos were used for RNA extraction and cDNA synthesis in each group. In all cases, mRNA expression of *pitx3, drd2a* and *drd2b* was normalized to the expression of *ef1α*, which was taken as the reference gene. A minimal variation in *ef1α* (Fig. 7G) expression was found in the 3'UTR-Pitx3 microinjection group with respect to the control group. Each bar represents the number of cDNA copies of the genes studied ± SEM. For each group, three experiments were performed, each in triplicate. *P* values were calculated with the two-tail unpaired Student's t test. *P<0.05, **P<0.01 and ***P<0.001.

miR-133b acts as a negative regulator of the expression of the Pitx3 transcription factor by binding to its 3'UTR region [31]. Pitx3 activates the transcription of the genes required for the differentiation of dopaminergic neurons, such as the synthesis of TH, DAT and dopamine receptors. Thus, in order to silence miR-133b, 750 pg of the DNA sequence of 3′UTR-Pitx3 was microinjected into the one-cell stage of zebrafish. At 24 hpf, we observed that microinjection of the 3'UTR-Pitx3 DNA sequence produced a consistent decrease of more than 10-fold (Fig. 7A). Likewise, *pitx3* expression, the miR-133b target, showed a significant upregulation, reaching a level that almost doubled the control group (Fig. 7B). *th* was upregulated by microinjection of the 3′UTR-Pitx3 DNA sequence (Fig. 7C), whereas *dat* (Fig. 7D) did not show consistent changes. Likewise, in the same direction than *pitx3* and *th*, the transcription levels of the *drd2a* receptor were increased (Fig. 7E); conversely, the expression of *drd2b* (Fig. 7F) was decreased almost two-fold. The results of our qPCR analyses were normalized based on the expression of *ef1a* (Fig. 7G), ever since this gene did not show any consistent changes following microinjection of the DNA sequence of 3′UTR-Pitx3.

## Discussion

Most of the published data addressing addictive drugs, as cocaine for example, have focused on adult or neonate models in mammals [53], [54]; very little information has been published concerning early embryonic development and addiction. In this sense, our research was addressed to analyse the effects of cocaine during several stages of embryonic development using zebrafish embryos, since this organism is considered a model for vertebrate development [55], [56]. Our aim was to determine the possibility that cocaine, via miR-133b, affects the expression of *pitx3* and its target genes (*th, dat* and dopamine receptors) in zebrafish embryos.

### Phylogeny of All Dopamine Receptors in Zebrafish

Our analysis revealed that the phylogenetic tree constructed from the amino acid sequences of dopamine receptors have a monophyletic origin (the five types of dopamine receptors result from a common gene) (Fig. 1A and B). The zebrafish dopamine receptors are aligned in two major families: the D1-like receptor (including *drd1*) and the D2-like receptor (formed by *drd2a, drd2b, drd2c, drd3, drd4a, drd4b*, *drd4rs*). Since the mammalian dopamine receptors families -D1-like and D2-like- have been shown to be coupled to G_s_α or G_i_α G-protein, respectively [48], [49], [55], our findings suggest that according to their phylogenetic alignment zebrafish dopamine receptors indicate which type of G protein they are coupled to (G_s_α or G_i_α) (Fig. 1A and B). Hence, each family of zebrafish dopamine receptors modulates different intracellular signalling pathways [57].

### Cocaine Affects the Expression of Dopamine Receptors

In the present research, in the early stages of embryonic development (8 and 16 hpf) of zebrafish we detected the presence of dopamine receptor mRNA (*drd1, drd2a, drd2b* and *drd3*) (Fig. 2). These results suggest that these receptors could be involved in the process of gastrulation (8 hpf), neuromere formation, and CNS differentiation (16 hpf). Our results differ from those reported in the study of Li et al. (2007) and Boehmler et al. (2004) [17], [18], who detected the presence of *drd1* at 30 hpf, *drd2a*, *drd2b and drd3* approximately at 15 hpf by ISH. We propose that the absence of dopamine receptors in early development detected by ISH [17], [18] does not mean that there is no dopamine receptor gene expression, since we found the expression of a few copies of cDNA (approximately between 20–400 copies of cDNA) by qPCR of *drd1, drd2a, drd2b* and *drd3* (Fig. 2). The accuracy of our qPCR analysis is consistent, since the results are based on the expression of the *ef1α* and *β-actin* reference genes. Also, these mentioned genes did not show consistent changes induced by cocaine with respect to their respective control group at the different development stages. Our findings are similar to those of previous studies that have also demonstrated that *ef1α* and *β-actin* undergo few variations in their expression during different zebrafish developmental stages and that such changes depend on the different chemical treatments applied [58]. In this sense, zebrafish embryos exposed to cocaine showed alterations in the expression of *drd1, drd2a* and *drd2b* during the early and late stages of embryonic development (16, and 24 hpf; 48 and 72 hpf, respectively), suggesting that early alterations would affect the proper development of their dopamine receptors and hence also their functions. Dopaminergic neurons appear between 18–22 hpf in zebrafish embryos [59], [60]. Thus, we consider that the changes observed in the expression of the dopamine receptors *drd1, drd2a, drd2b* and *drd3* in the early stages of embryonic development could be due to the effects of cocaine on the expression of several transcription factors, such as Lmx1b, Otpa, Nurr1 (unpublished results) and Pitx3, because these factors are important in the formation of the dopaminergic neuronal precursors and their differentiation [61], [62], [63], [64], [65], [66].

### Cocaine Affects the Expression of miR-133b and *pitx3* and its Target Genes

The Pitx3 transcription factor, expressed in the CNS and also in muscle [51], [52], is required for the development and survival of midbrain dopaminergic neurons [67], [68] and miR-133b has been associated with Pitx3 regulation, binding specifically to 3'UTR-Pitx3 (in mammals [31] and zebrafish [69]). Moreover, it has been described that miR-133b regulates the maturation and function of midbrain dopaminergic neurons within a negative feedback loop including Pitx3 [31], where the Pitx3 transcription factor induces miR-133b expression and miR-133b decreases Pitx3 activity post transcriptionally [31]. To our knowledge, this is the first time that the spatial distribution of miR-133b has been studied in zebrafish. This miRNA exhibited an important level of expression in somites as well as a weak expression in the diencephalon, midbrain and hindbrain. At 48 hpf we observed that the expression of miR-133b was more intense in the region of somites, unlike at 24 hpf, where it was less marked. The higher expression of miR-133b in somites indicates that it must play a crucial role in the proper development of skeletal muscle [70]. In addition, we also observed a localized expression of miRNA in the diencephalon, midbrain and hindbrain, following the pathway of the median longitudinal fasciculus. Although the expression of miR-133b was less intense in the CNS at 24 and 48 hpf as compared to its expression in somites, this level of expression could be sufficient to play a relevant role in the development and function of the CNS in zebrafish. In this sense, our results suggest that this miRNA could be essential for morphogenesis, brain formation, neural differentiation, and somitogenesis, among other processes, during zebrafish embryonic development [67], [68].

The exposure of zebrafish embryos to cocaine did not reveal evident changes by ISH in the expression of miR-133b in the brain and at the periphery at 24 and 48 hpf. Thus, to determine the expression of miR-133b both in the CNS and at the periphery we decapitated the embryos at 24 and 48 hpf of embryonic development. We observed by qPCR that miR-133b was expressed in higher amounts at the periphery (Fig. 4M) than in the CNS (4G). These results are consistent with our ISH analyses of miR-133b (Fig. 5A and 6A). Accordingly, the major changes observed in our qPCR experiments (Fig. 4A) occurred at the periphery (Fig. 4M), although miR-133 also displayed changes in the CNS to a lesser extent (Fig. 4G). These changes may be enough to produce changes in the expression of its target, Pitx3. These results, at 24 and 48 hpf, indicate that a decrease of miR-133b in the CNS induce an increase of its putative target gene, *pitx3*. Consequently, the upregulation of *pitx3* induced an increase of the dopamine phenotype markers: *th, drd2a* and *drd2b* (Fig. 4I, J, K and L, respectively) (except *drd2b* and *dat* at 48 hpf; Fig. 4J and L, respectively). Likewise, at 24 hpf our observations showed an increase in the expression of zebrafish dopamine receptors *drd2a* (in the epiphysis and tegmentum), *drd2b* (hindbrain) and *drd3* (tegmentum). Therefore, an upregulation in the expression of dopamine phenotype markers induced by cocaine will induce an increase in the number of dopaminergic neurons; an example is the increase in the tyrosine hydroxylase activity in the brain [71], [72], although it has also been described that cocaine may decrease the number of dopaminergic neurons [73], [74]. It is remarkable to see that cocaine induced, in whole-mount embryos, different effects on the expression of miR-133b at 24 and 48 hpf (increase and decrease, respectively). The decrease of miR-133b (Fig. 4A) was correlated with an increase of *pitx3* at 24 hpf, surprisingly the same effects were not seen at 48 hpf; a decrease of *pitx3* was found (Fig. 4B). When the embryos were decapitated, the expression of *pitx3* was observed in higher amounts at in the periphery than in the CNS, and the pattern of expression was similar to that seen in whole-mount embryos. Conversely, in the CNS *pitx3* expression was low, but an increase in its expression was observed at 24 and 48 hpf. These results indicate that changes in the expression of *pitx3* (at 48 hpf) in the CNS (Fig. 4H) are masked by its expression at the periphery (Fig. 4N), when whole mount embryos are considered (Fig. 4B). It remains unclear how *pitx3* has different profiles in its expression in the CNS and at the periphery.

### miR-133b Alters the Expression of *pitx3* and its Target Genes

The aim of microinjection of 3'UTR-Pitx3 was to increase the excess of targets that interact with miR-133b and thus silence its expression. In this way, endogenous *pitx3* mRNA is increased, producing more than its physiological expression [69]. In the present study, when embryos were injected with the target of miR-133b (the 3′UTR-Pitx3 sequence) we found a downregulation of miR-133b (Fig. 7A) levels and an increase in *pitx3* (Fig. 7B), *th* and *drd2a* (Fig. 7C and E, respectively) transcript levels. This indicates that miR-133b is regulating the actions of *pitx3* and its target genes in zebrafish embryos. These results are similar to those found by Sánchez-Simón et al. [69] in the regulation of *pitx3* by miR-133b. It is interesting to note that a clearly different expression was observed by qPCR of *drd2a* and *drd2b* after exposure to cocaine (Fig. 2B and 2C, respectively) in whole-mount embryos and after 3'UTR-Pitx3 DNA microinjection (Fig. 7E and F, respectively). The opposite changes in the expression of *drd2a* and *drd2b* in whole-mount embryos are not surprising, since *drd2a* and *drd2b* are duplicate genes in the zebrafish genome, with different distributions at 24 and 48 hpf [18], and duplicate genes may have individual actions as compared with their ancestral gene [75], [76]. This observation is similar to what has been reported for other zebrafish duplicate genes, such as the two duplicates for delta opioid receptors in zebrafish. These receptors show a different spatial and temporal profile expression during zebrafish development [77] and also display different pharmacological properties [78], in spite of the fact that they share a good homology between one another (71%) [78], [79].

**Figure 8:**
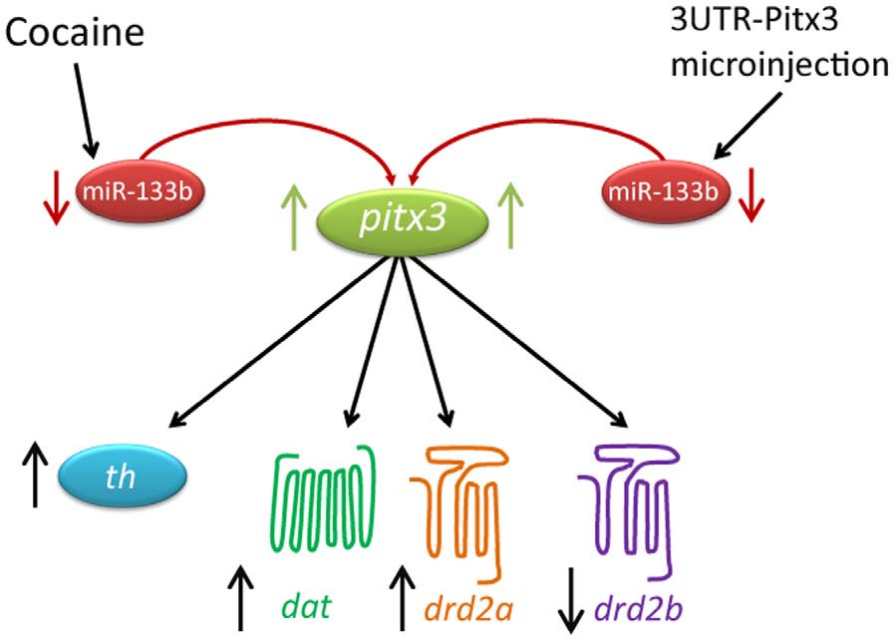
Possible mechanism of action of cocaine through miR-133b in zebrafish embryos. In the present study, both microinjection of 3′UTR-Pitx3 sequence (which simulates the silencing of miR-133b) and exposure to cocaine, in embryos of zebrafish, induced a decrease of miR-133b expression. This alteration of the expression of miR-133b induced an increase of *pitx3* and its targets genes: *th, dat, drd2a*, while the expression of *drd2b* was decreased (it should be noted that cocaine produced an increase of its expression in the encephalon). Our findings support the existence of a negative regulation between miR-133b and *pitx3* in zebrafish embryos, since a downregulation of miR-133b produced an upregulation of *pitx3*, *dat, th* and *drd2a*. These results lead us to propose that cocaine via miR-133b may affect the development and the function of the dopaminergic system.

Our *in vivo* findings (at 24 hpf of zebrafish development) showed that the exposure to cocaine produced a decrease in miR-133b expression in a manner similar to 3'UTR-Pitx3 microinjection, which increased the Pitx3 transcription level (due to a negative regulation between them), and hence the expression of the target genes of Pitx3 was altered, producing an upregulation of *th, dat* and *drd2a* in whole-mount embryos. In this sense our findings lead us to propose a possible mechanism of cocaine action, on the regulation of *pitx3* and its target genes, through miR-133b (Fig. 8). Thus, the changes produced by cocaine in the expression of miR-133b could alter the development of the dopaminergic system, at the periphery and in the CNS, during embryonic development of the zebrafish. This new insight into the actions of cocaine opens a door to the design and synthesis of specific drugs that could inhibit the activity of miR-133b to prevent or compensate the effects of cocaine and avoid possible damages in the development of the CNS in the embryos exposed to cocaine.

### Conclusions

The present study using zebrafish embryos suggests that by altering the expression of miR-133b, cocaine affects the expression of its target gene *pitx3*. When the expression of the *pitx3* transcription factor changes, its target genes (*th*, *dat, drd2a and drd2b* receptors) also subsequently follow the same trend as *pitx3*. Likewise, the cocaine-induced changes of miR-133b and its target gene, *pitx3,* depend on the stages of zebrafish embryonic development. Thus, using the zebrafish as a developmental vertebrate animal model can shed light on the mechanism(s) that at molecular level regulates the effect of cocaine, throughout the different stages of embryonic development.

## Materials and Methods

### Ethics Statement

All procedures and experimental protocols were carried out in accordance with the guidelines of the European Communities Council directive of 24 November 1986 (86/609/EEC), current Spanish Legislation (BOE 67/8509-12, 1998) and following the Guide for the Care and Use of Laboratory animals as adapted and promoted by the US National Institute of Health.

All experiments were performed at the University of Salamanca with the approval of the University of Salamanca Animal Care Committee.

### Animals

Wild-type zebrafish embryos (*Danio rerio*, wild-type AB strains) were used in this study and staged according to development in hours post-fertilization (hpf) according to Kimmel et al (1995) [80]. To obtain these embryos, adult zebrafish were raised under a 12 h light: 12 h dark (LD) cycle at 26°C in a multi-tank system at the Fish Facilities of the University of Salamanca, simulating their environmental conditions. Fertilized embryos were selected using a stereomicroscope (Leica Z2000, Nussloch, Germany), raised at 28.5°C, and maintained in dishes containing sterile E3 medium (5 mM NaCl, 0.17 mM KCl, 0.33 mM CaCl, 0.33 mM MgSO4 in distilled water; Sigma, Madrid, Spain). All efforts were made to minimize the number of embryos used and their suffering.

### Drug Treatment

Cocaine HCl was administered to the embryos diluted in E3 medium, their aqueous environment, at a concentration of 1.5 µM [20], [21] at the 5 hpf stage. First, for the dopamine receptors -*drd1, drd2a, drd2b* and *drd3*- we studied the effect of cocaine at the 8, 16, 24, 48 and 72 hpf of embryonic developmental stages to see whether it produced any change, then, we chose 24 and 48 hpf stages to analyse the *pitx3* transcription factor and miR-133b since both stages are essential in embryonic development, 24 hpf determines the end of the segmentation period, during which the CNS (Central Nervous System) is being formed and differentiated, and 48 hpf defines the end of organogenesis [80].

The concentration of cocaine HCl (1.5 µM) chosen is comparable to the concentration present in human maternal and foetal serum, placental fluid and foetal urine [81] and in human umbilical cord in neonates [82]. The doses of 1.5 µM cocaine HCl was chosen from the three doses studied (0.5, 1.5 and 3 µM), since it induced consistent changes in the expression of most genes studied and also considering the fact that this dose did not produce anaesthetic effects in adult zebrafish [21].

### Determination of Cocaine Entering Zebrafish Embryos by High-performance Liquid Chromatography-mass Spectrometry (HPLC-MS)

Zebrafish embryos were exposed to 1.5 µM cocaine-HCl from 5 to 24 hpf and from 5 to 48 hpf, after which they were harvested. Embryos at both developmental stages were washed three times for 5 min each in E3 medium and dechorionated. Dechorionation was performed in order to quantify the real cocaine concentration in embryonic tissue, since the chorion might prevent cocaine from entering the embryo. Embryos were kept at 20°C. Samples were defrosted first at 4°C (1 h) and then at room temperature. After adding 1 ml of 10 mm ammonium formate, pH 9.3 [83], samples were mechanically homogenized with a Polytron device on ice. Homogenized embryonic tissue was centrifuged for 30 min at 4000 g at 4°C and the supernatants were collected and kept at 4°C [83] until HPLC-MS analysis. Six samples per developmental stage (200 embryos were used per sample) were analysed by HPLC-MS. HPLC-MS analyses were performed as previously described [83] using a Waters ZQ 4000 device with an Alliance HT HPLC apparatus. HPLC conditions were as follows: column, Atlantis T3, 3 µm, 2.1×100 mm; solution A, 10 mm ammonium formate, pH 7.0, in H_2_O; solution B, methanol. Initial conditions were 30% B and a gradient was performed over 11 min to reach 100% B. The 286 and 289 u.m.a. signals were recorded and integrated in SIM mode. Cocaine-D was used as an internal deuterated standard (Cerrilliant, Round Rock, TX, USA). Using the described methodology, 0.175±0.0472 nm of cocaine was detected in each embryo, which represents approximately 12% of the initial concentration of the 1.5 µM cocaine used.

### Decapitation of the Embryos

To study the expression of several genes in whole-mount zebrafish embryos, these were exposed to Trizol reagent (Invitrogen) at 24 and 48 hpf. Upon studying gene expression only in the head and at the periphery (the rest of the body without the head), zebrafish embryos were decapitated at 24 and 48 hpf. Thus, to minimize suffering and distress in zebrafish embryos, the embryos were first immobilized by immersion in an anaesthetic solution: 0.016% tricaine (Ethyl 3-aminobenzoate methanesulfonate salt/MS-222 in E3) at a pH between 7.2 and 7.4 for at least 2 minutes. Following cessation of the movement of the embryos to touch, using an insulin syringe (30 Gauge, 1cc, 5/16″) embryos were rapidly decapitated. Each needle of the insulin syringe was crossed at the limit between the separation of the hindbrain and the spinal cord at 24 hpf (at the level of the otic vesicle) and at 48 hpf (at the level of the pectoral flipper). Then, the needles were pulled in the opposite direction. After decapitation, the head and the rest of the body were collected immediately.

### RNA Extraction and DNase I Treatment

Zebrafish embryos were divided into two groups, a control group and a cocaine-treated group, for each stage of study. Two hundred and fifty embryos were used per group. Total RNA, including miRNA, was extracted using Trizol reagent (Invitrogen Corp. Carlsbad, CA, USA) following the protocol recommended by the manufacturers. In all cases, the RNA samples were treated with DNase I (Roche Scientific, Madrid, Spain) following the protocol recommended by the manufacturers. RNA concentrations were determined using NanoDrop technology (NANODROP 2000C spectrophotometer, Thermo Scientific). Each RNA sample was assayed three times and a mean value was determined for the experiments.

### Retrotranscription and RNase Treatment

cDNA synthesis was carried out by reverse transcription of total RNA to cDNA using the Promega Corp. (Madison, WI, USA) reverse transcription kit. The RNA was combined with dT oligonucleotide in a total volume of 5 µl and incubated at 70°C for 5 min. The final volume of each reaction was brought up to 20 µl by the addition of 4 µl of retrotranscriptase, 3 µl of MgCl_2_ and 5.5 µl of diethyl pyrocarbonate-treated water. Samples were then incubated for 10 min at 25°C, followed by 1 h at 42°C and 15 min at 70°C. NCode miRNA First-Strand cDNA Synthesis (Invitrogen Corp. Carlsbad, CA, USA) was used to synthesize cDNA from miRNA following the protocol recommended by the manufacturers. All the cDNA samples obtained were then treated with RNase A (20 ng/µl for 7–8 µg cDNA) for 20 min at 37°C. Since cDNA is a (DNA–RNA) double-stranded hybrid, RNase A does not act on these molecules. The product was purified using QIAquick PCR Purification Kit columns from QIAGEN and eluted in DNase-free water. The cDNA concentration was determined by measuring absorbance at 260 nm with NanoDrop technology.

### Quantitative Real Time PCR

Quantification of the PCR (qPCR) products was accomplished with a standard curve using the SYBR-Green method. The SYBR- Green was included in a 2× Master Mix from Applied Biosystems (Alcobendas, Madrid, Spain) (SYBR Green dye, dNTPs, Passive Reference (ROX), Amplitaq1 Gold DNA polymerase). The oligonucleotides used to amplify the genes studied in this work were as follows: *ef1α*: forward, GTACTTCTCAGGCTGACTGTG; reverse, ACGATCAGCTGTTTCACTCC. *β-actin:* forward, ACCACGGCCGAAAGAGAA; reverse, ATACCCAGGAAGGAAGGCTG. *drd1*: forward, ACGCTGTCCATCCTTATCTC; reverse, TGTCCGATTAAGGCTGGAG. *drd2a*: forward, TGGTACTCCGGAAAAGACG; reverse, ATCGGGATGGGTGCATTTC. *drd2b*: forward, AAATAACACAGCTACACGGGAT; reverse, GAACCACGTAAATCTGCACG. *drd3*: forward, ATCAGTATCGACAGGTATACAGC; reverse, CCAAACAGTAGAGGGCAGG. *pitx3*: forward, GACAACAGTGACACAGAGAAGT; reverse, GAGAAACCGTTATCCCGACA. *th*: forward, TTTGAAGAGAAGTGCAGAGGAT; reverse, GGATCACCCAGGATTTACTGA. *dat*: AGACATCTGGGAAGGTGGTG; reverse, ACCTGAGCATCATACAGGCG. *dre-miR-133b*: forward, TTTGGTCCCCTTCAACCAGCTA; reverse Universal qPCR Primer (oligo from NCodeTM miRNA First-Strand cDNA Synthesis and qRT-PCR Kits) (Invitrogen). The ABI Prism 7300 detection system (Applied Biosystems) was used to amplify the different genes under the following conditions: 10 min at 95°C followed by 36 cycles of 15 s at 95°C and 1 min at 55–60°C (*ef1α* and *β-actin,* 55°C*; drd3,* 56°C*; th, pitx3* and miR-133b, 57°C*; drd2a,* 59°C*; drd1* and *drd2b,* 60°C). A minimum of three PCR reactions was performed for each sample per plate, and each experiment was repeated three times. The final volume of each reaction was 20 µl: 10 µl of Master Mix, 1 µl of each oligonucleotide, 7 µl of distilled water and 1 µl of cDNA at a concentration of 5 ng/µl. *ef1α* was used as a gene reference.

Standard curves were obtained for absolute quantification of the several genes exposed to cocaine with respect to the control group. First, a standard PCR was carried out to amplify the fragment of the transcript of interest (*drd1, drd2a, drd2b, drd3, pitx3* and miR-133b) on zebrafish cDNA and the same pair of primers was used for the qPCR experiments. The fragment was cut from the agarose gel and purified. Serial 1∶10 dilutions were made from the purified PCR product, ranging from 10^−2^ ng/µl to 10^−5^ ng/µl, which afforded a four-point straight line that had a slope of approximately −3.3.

### Cloning and Microinjection of 3'UTR-Pitx3

3′UTR from Pitx3 was amplified using specific primers based on the sequence of the full-length cDNA from Ensemble (accession number ENSDARG00000070069) using the TaKaRa LA Taq™ kit. The following primers were used: *pitx3* 3′UTR: forward, CGGTATGAAAGCGATGCGTCTA; reverse, TCCTGGTGTAGCCTGCTTTGTCT. The program used for amplification was as follows: 5 min at 95°C followed by 35 cycles of 15 s at 95°C, 30 s at 57°C, and 1 min at 70°C. At the end of the cycles, a final extension temperature of 70°C was added for 10 min. The PCR products were purified and cloned into pCR®II vector (Invitrogen). TOP 10`F cells (Invitrogen) were transformed with the construct. This construct was digested with EcoRI for 1 h at 37°C, after which it was purified and sent for sequencing.

### Microinjection of the 3'UTR-Pitx3 Clone

After corroboration of the sequence, the amplicon of 723 nucleotides was injected at a concentration of 250 ng/µl into single-cell zebrafish embryos with a micromanipulator-microinjector system from Eppendorf AG (Hamburg, Germany). The aim of microinjection of the target of miR-133b (3′UTR-Pitx3) was to increase the excess of targets that interact with miR-133b and thus silence its expression.

### Whole-mount *in situ* Hybridization

Embryos at 24 hpf and/or 48 hpf were dechorionated, fixed in 4% paraformaldehyde (PFA)/phosphate buffer saline (PBS) overnight at 4°C, then washed twice in PBS, at room temperature (RT), and finally maintained in absolute methanol at −20°C until use. Embryo rehydration was accomplished by successive dilutions of methanol/PBS (75, 50 and 25%). Then, the embryos were treated with Proteinase K (10 µg/ml) at RT for 15 min in 24-hpf and 40 min in 48-hpf embryos. Next, the embryos were refixed with 4% PFA/PBS, after which several washes with PBTw (PBS+0,1% Tween-20) were performed to remove residual PFA. The embryos were prehybridized for 2 h with the hybridization solution without probe. Then, the digoxigenin-labeled oligoprobe was added (10 ng/µl) and hybridization was carried out with the miR-133b riboprobe (miRCURY LNA™ microRNA Detection Probes for *in situ* hybridization, Exiqon) or dopamine receptor riboprobe. This was left overnight at 64°C (in the case of miR-133b) and 65°C (in the case of dopamine receptors) to hybridize. Two-hour washes with prehybridization solution/Tris buffer saline (TBS) (50%/50%) were performed, after which the embryos were blocked with blocking buffer (goat serum+TBST (TBS+0,1% Tween-20)) for 2 h and incubated overnight with the antidigoxigenin antibody conjugated with alkaline phosphatase (1∶3000; Roche) at 4°C. Then, the embryos were rinsed with Xpho solution (1 M Tris HCl, pH 9.5, 1 M MgCl_2_, 4 M NaCl and 20% Tween-20) three times. The DIG-AP antibody bound was detected with NBT/BCIP (Roche).

### Phylogenetic Analysis of Zebrafish Dopamine Receptors

The amino acid sequences from the dopamine receptors -*drd1, drd2a, drd2b* and *drd3*, orthologues of different vertebrate species- were aligned with the ClustalW program using default parameters (http://www.ebi.ac.uk/Tools/msa/clustalw2/). We conducted a phylogenetic analysis of all these orthologues of these zebrafish dopamine receptors using the Neighbour-Joining (NJ) method [43] and the Maximum Likelihood (ML) method [44]. The evolutionary analyses were conducted using MEGA5 [84].

### Image Analysis

The images obtained with whole-mount *in situ* hybridization were obtained with a Leica stereoscope (Zeiss) at 5, 6 and 10X magnification. All images were processed with Photoshop CS5 software (Adobe System Inc.) following the guidelines established by the Rockefeller University Press.

### Statistical Analyses

The real-time PCR results are represented as means ± SEM and pairs of the data were analysed by Student’s t-test. Multiple comparisons were evaluated by one-way and two-way ANOVA followed by Dunnett's and Bonferroni's post-hoc tests, respectively. In all tests, differences were considered significant at *P*<0.05. Statistical analyses were performed with GraphPad Prism 5 (GraphPad Software Inc. for windows, San Diego, CA, USA).

## Supporting Information

Figure S1 Doses effects of cocaine on the expression levels of *miR-133b*, *pitx3* and its targets. Expression of miR-133b (A), *pitx3* (B) and its targets genes *th, dat, drd2a* and *drd2b* (C, D, E and F, respectively) at 24 hpf in whole-mount embryos. Total RNA was isolated from two hundred and fifty embryos and used to synthesize cDNA. Expression of each gene (measured by qPCR) was normalized to *ef1α* expression. Error bars represent means (of mRNA copies at each developmental stage) ± SEM. Data are representative of three independent experiments and each experiment was performed three times. *P* values were calculated using one-way ANOVA followed by a post-hoc Dunnett*'*s test: *P<0.05, **P<0.01 and ***P<0.001.
(TIF)
