# Supplementary figures and images for: Modulation by Cocaine of Dopamine Receptors through miRNA-133b in Zebrafish Embryos
Source: PLoS One. 2012 Dec 21;7(12):e52701. doi: 10.1371/journal.pone.0052701 (PMC3528707; doi:10.1371/journal.pone.0052701)

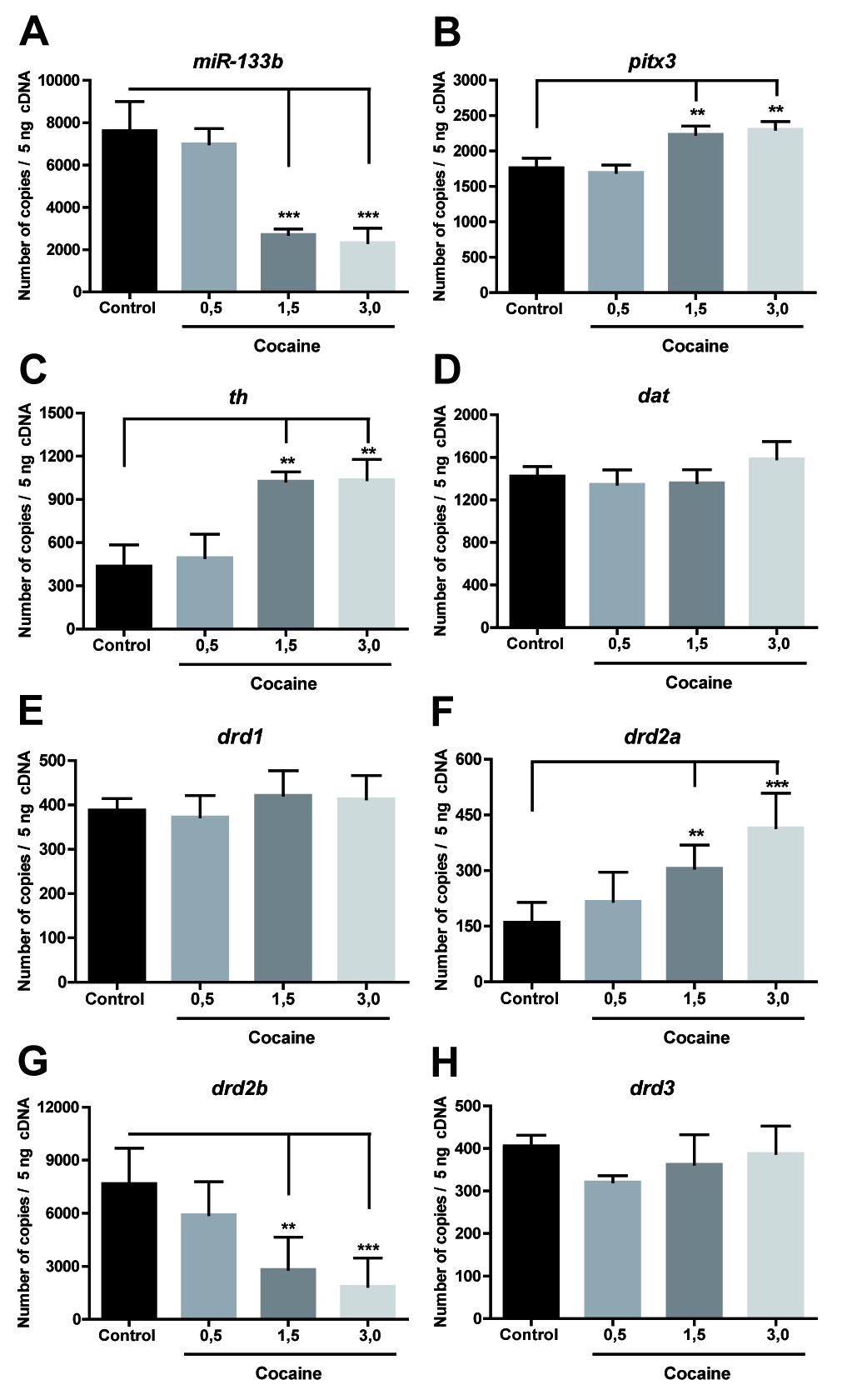

Supplement: Figure S1 — Doses effects of cocaine on the expression levels of miR-133b, pitx3 and its targets. Expression of miR-133b (A), pitx3 (B) and its targets genes th, dat, drd2a and drd2b (C, D, E and F, respectively) at 24 hpf in whole-mount embryos. Total RNA was isolated from two hundred and fifty embryos and used to synthesize cDNA. Expression of each gene (measured by qPCR) was normalized to ef1α expression. Error bars represent means (of mRNA copies at each developmental stage) ± SEM. Data are representative of three independent experiments and each experiment was performed three times. P values were calculated using one-way ANOVA followed by a post-hoc Dunnett's test: *P<0.05, **P<0.01 and ***P<0.001. (TIF) [file pone.0052701.s001.tif]
